# Partial mastectomy using manual blunt dissection (MBD) in early breast cancer

**DOI:** 10.1186/s12893-015-0102-5

**Published:** 2015-10-22

**Authors:** Shinichiro Kashiwagi, Naoyoshi Onoda, Yuka Asano, Kento Kurata, Tamami Morisaki, Satoru Noda, Hidemi Kawajiri, Tsutomu Takashima, Kosei Hirakawa

**Affiliations:** Department of Surgical Oncology, Osaka City University Graduate School of Medicine, 1-4-3 Asahi-machi, Abeno-ku, Osaka, 545-8585 Japan

**Keywords:** Breast cancer, Manual blunt dissection, Partial mastectomy, Surgery, Breast-preserving surgery

## Abstract

**Background:**

Breast-preserving surgery (Bp) and sentinel lymph node biopsy (SNB) are established as standard treatment for axillary lymph node-negative early breast cancer.

**Methods:**

A surgical technique using manual blunt dissection (MBD), in which use of electrocautery, an ultrasonically activated scalpel, and ligation is minimized, is described. This involves an approach from small incisions in the axilla or areola to avoid injury to skin flaps, and with adequate mobilization of the breast, so that regardless of the tumor site, surgical wounds are not noticeable. The usefulness and tolerability of this surgical technique were examined.

**Results:**

This surgical technique was evaluated in 233 patients. Surgery could be performed rapidly, with a mean operative time of 67 ± 21 min and a low mean blood loss of only 35 ± 28 ml. There was little need for postoperative analgesia, and surgery was well tolerated without postoperative bleeding or wound infection.

**Conclusion:**

Our proposed technique for partial mastectomy using MBD provides good curative and cosmetic results.

## Background

Breast-preserving surgery (Bp) and sentinel lymph node biopsy (SNB) are established as standard treatment for axillary lymph node-negative early breast cancer [1–3]. With the addition of appropriate postoperative radiotherapy, survival rates equivalent to mastectomy, low recurrence in the preserved breast, and acceptable treatment outcomes have been achieved [4–6]. New surgical techniques to maintain these cure rates and provide good cosmetic results must now be developed.

A surgical technique using manual blunt dissection (MBD), in which use of electro cautery, an ultrasonically activated scalpel, and ligation is minimized, is described. It involves an approach from small incisions in the axilla or areola to avoid injury to skin flaps, and with adequate mobilization of the breast, so that regardless of the tumor site, surgical wounds are not noticeable. The usefulness and tolerability of this surgical technique were examined.

## Methods

### Patient characteristics

This study included patients with primary breast cancer that was confirmed preoperatively by needle biopsy between January 2010 and December 2012. Surgical outcomes for 233 patients who underwent Bp + SNB at our hospital are shown. The indications for this surgery were a preoperative diagnosis of ductal carcinoma in situ (DCIS) or tumor size ≤3 cm for invasive carcinoma, each without clinical axillary node metastases (cN0) [2]. Lesion extent was assessed by ultrasound and magnetic resonance imaging (MRI). For the mammary excision stump, a tumor margin that was less than 5 mm was considered positive and, in positive cases, a radiation boost of 10 Grays was added. In addition, when there was marked denudation of the cancer nest, re-excision was performed. Cosmesis was evaluated after Bp using a patient-based tool, the “Four points of evaluation of Harvard” [7].

Axillary clearance was indicated for SN metastasis, defined as macrometastasis (i.e., tumor diameter >2 mm) in the SN. The presence of micrometastasis (i.e., tumor diameter >0.2 mm, ≤2 mm, or <200 tumor cells) or isolated tumor cells (ITCs, i.e., tumor diameter <0.2 mm or <200 tumor cells) was not considered an indication for axillary clearance [8].

This study was performed in accordance with the Declaration of Helsinki and carried out with the approval of the Ethical Review Board of Osaka City University (#926). A sufficient explanation was provided, and written, informed consent was obtained from all study subjects for their involvement in this study and for the storage and use of their data.

### Surgical procedure

**Fig. 1:**
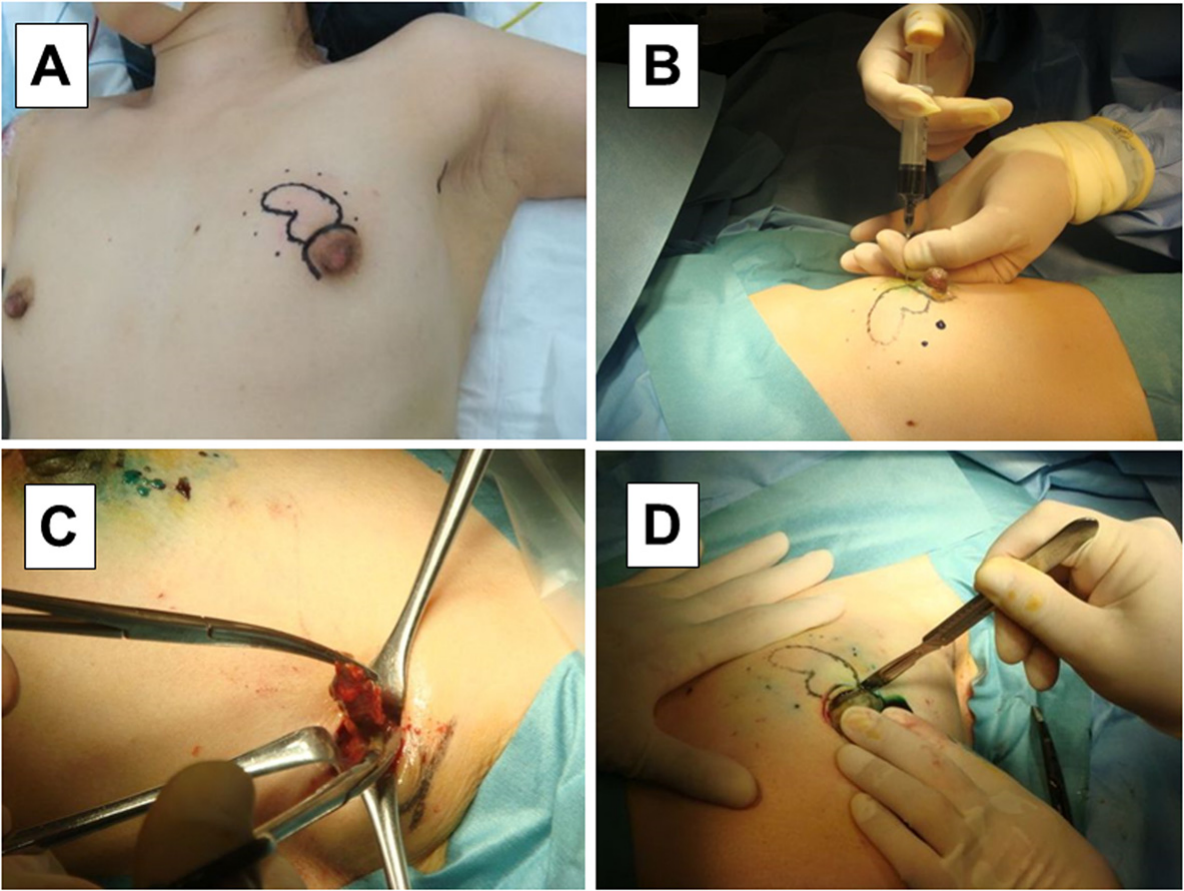
Surgical procedure of MBD (I). The target tumor was confirmed by palpation and ultrasound before surgery (**a**). Lidocaine jelly mixed with indigo carmine was percutaneously injected (**b**). A 2-cm skin incision was made into the axilla for SNB (**c**). A skin incision was made in the areola to perform a partial mastectomy (**d**)

The target tumor was confirmed by palpation and ultrasound before surgery (solid line), and markings were made at 1-cm margins on the skin directly above (dotted line) the planned resection site (Fig. 1a). Lidocaine jelly mixed with indigo carmine was percutaneously injected along the markings (dotted line) into the breast surface of the mammary gland (Fig. 1b), and about 40 mL of 1 % lidocaine + epinephrine (3-fold dilution) were injected subcutaneously around the planned area of dissection for the purpose of hemostasis.

First, a 2-cm skin incision was made into the axilla for SNB, and the sentinel lymph node (SN) was sent fresh for intraoperative histopathology (Fig. 1c). The SN was identified using radioisotope (RI) and staining methods [9–13]. For the RI method, 1 millicurie (mCi)/mL of 99 m-technetium (Tc)-phytate colloid was injected intradermally and subcutaneously directly above the tumor and around the tumor on the day before surgery [14–16]. Lymphoscintigraphy was then performed to identify a possible SN outside the axilla and search for the SN during surgery. For the staining method, 5.0 mL of indocyanine green (Diagnogreen; Daiichi Sankyo, Tokyo, Japan) were injected subcutaneously to make a wheal in the areola of the affected breast [17]. The area around the injection was lightly massaged, and about 10 min later, a skin incision was made to identify a green-stained lymph node as the SN. The SN was removed, cut into 2-mm-thick sections, and histopathologically examined by an experienced pathologist. Only macrometastases were considered positive, whereas micrometastases and ITCs were considered negative [8, 18, 19]. Patients with a positive SN underwent further axillary lymph node dissection [20].

**Fig. 2:**
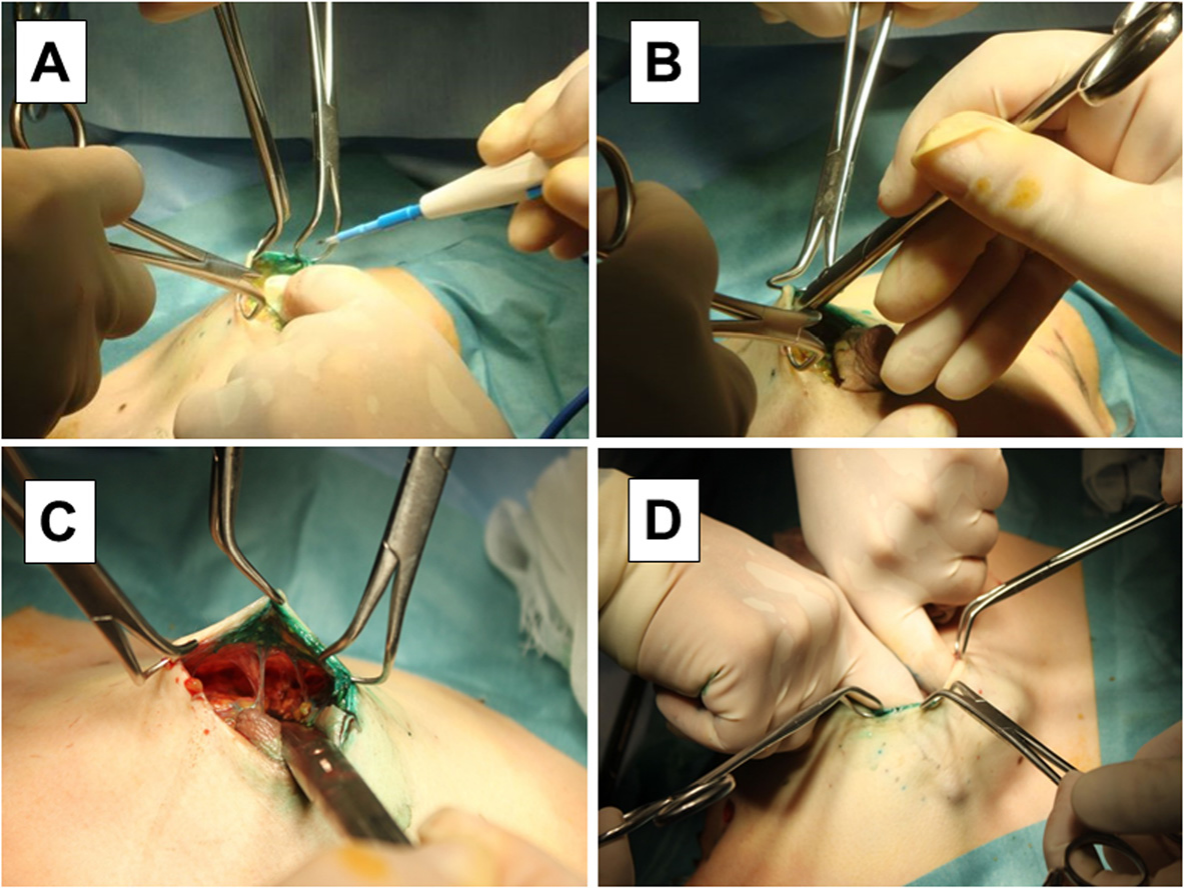
Surgical procedure of MBD (II). The skin around the wound margin was grasped with sharp towel clamps (**a**). Blunt subcutaneous dissection was performed using curved Cooper scissors (**b**). Using counter traction with the towel clamps, the subcutaneous tissue was dissected (**c**). The breast in the planned area of dissection was completely mobilized from the skin (**d**)

Next, a skin incision was made in the areola to perform a partial mastectomy (Fig. 1d). A crescent-like incision was made to expose the surgical field (an arc-like incision was made in the breast margin for the tumor in the outer upper region, and SNB + Bp were performed from the same surgical wound). The skin around the wound margin was grasped with sharp towel clamps, and a 1 to 2-mm length of skin and subcutaneous fat was dissected by electro cautery (Fig. 2a). Blunt subcutaneous dissection was performed using curved Cooper scissors. The Cooper scissors were held with the tip toward the skin side, and, with the tip closed, the tip was slid along the back of the skin and pushed (Fig. 2b). Dissection of the planned area, including directly above the tumor, proceeded in various directions. Using counter traction with the towel clamps, the subcutaneous tissue was dissected, being careful to avoid pulling of the breast skin (Fig. 2c). With the procedure thus far, only Cooper’s ligament remained, so Cooper’s ligament was manually dissected, and the breast in the planned area of dissection was completely mobilized from the skin (Fig. 2d).

**Fig. 3:**
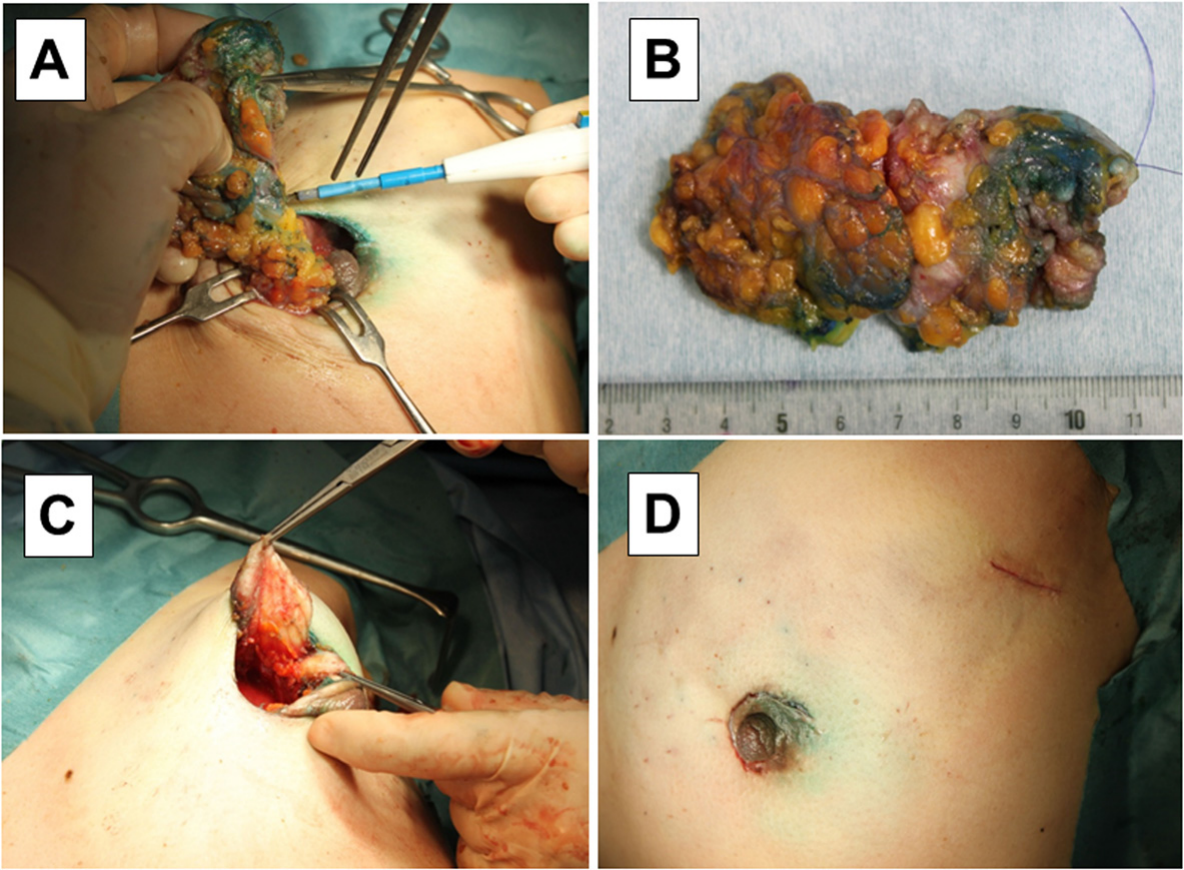
Surgical procedure of MBD (III). Removing the breast tissue through the surgical wound (**a**). The resected tumor specimen was confirmed, retention sutures were placed on the nipple side (**b**), and the breast margins were sutured and closed **(c)**. The wound was closed with buried subcuticular sutures (**d**)

The breast was incised vertically along the markings near the areola until the retromammary space was reached, and then the retromammary space was manually dissected. This area was widely dissected to facilitate breast flap mobilization after resection. While removing the breast tissue through the surgical wound, dissection proceeded along the markings for breast tumor resection (Fig. 3a). Retention sutures were attached to the resected tumor specimen so that the pathologists could understand which was the nipple side at the time of pathological diagnosis (Fig. 3b), and the breast margins were sutured and closed (Fig. 3c). If any skin was being pulled during suture closure, further subcutaneous dissection in this area was performed to avoid pulling. The wound was closed with buried subcuticular sutures and cyanoacrylate adhesive applied to the skin. In principle, no drains were placed (Fig. 3d).

## Results

This surgical technique was evaluated in 233 patients. Surgery could be rapidly performed, with a mean operative time of 67 (range 42–126, median 68, standard deviation 21) min and a low mean blood loss of only 35 (range 1–136, median 33, standard deviation 28) ml. Breast reconstruction by lateral tissue flap (LTF) was done in 18 cases (7.7 %). Re-excision was performed in five cases (2.1 %) to save tumor stump exposure. Macrometastasis was detected in 31 patients (13.3 %), and all of these patients underwent axillary lymph node dissection. Micrometastasis was observed in 20 patients (8.6 %). Cosmesis was evaluated as “Good” or better by 198 cases (85.0 %) (Excellent *n* = 86, Good *n* = 112, Fair *n* = 29, Poor *n* = 6). There was little need for postoperative analgesia, and surgery was well tolerated, with little postoperative bleeding or wound infection (hematoma *n* = 3, wound infection *n* = 1) (Table 1).

**Table 1:** Patient background and operation outcomes

Parameters	Bp + SNB (n = 233)
Age (years)	mean 59 (range 30–95, median 59, standard deviation 13)
Tumor diameter (cm)	mean 2.0 (range 0.4–3.8, median 2.0, standard deviation 0.8)
Operation time (min)	mean 67 (range 42–126, median 68, standard deviation 21)
Estimated blood loss (ml)	mean 35 (range 1–136, median 33, standard deviation28)
Frequency of total analgesics	mean 2.5
Cosmesis (Four points of evaluations of Harvard)	Excellent 86, Good 112, Fair 29, Poor 6
Postoperative bleeding	3
Wound infection	1

*Bp* breast-preserving surgery, *SNB* sentinel lymph nodes biopsy

## Discussion

Bp is minimally invasive and now considered standard treatment for early breast cancer [1–3]. However, with a partial mastectomy for tumor resection, some degree of breast deformity is difficult to avoid, and patients may be concerned about surgical scars of the breast skin. We use an incision of the anterior axillary line or areola to preserve skin directly above the tumor. In addition, for any deformity due to breast resection, breast reconstruction using residual breast and adipose tissue, or simultaneous breast reconstruction using an autologous LTF or latissimus dorsi muscle flap (LDMF) can be performed. This has provided excellent cosmetic results [21, 22]. The results of the present study apply to the small breasts of Japanese women, but, in the case of larger breast patients or patients with ptosis, MBD may be difficult when considering the distance from the skin incision to the tumor.

Electrocautery and thermal cautery were previously used for flap creation, but this often caused flap skin damage or burn. However, MBD using Cooper scissors can be used to quickly create a wide flap without skin damage and thus reduce operative time. With wide flap creation and adequate dissection of the retromammary space, an axillary or areolar approach can be used for an inconspicuous surgical scar, regardless of the tumor site. Our surgical technique enables breast flap reconstruction without skin pulling to provide excellent cosmetic results. In the present study, general anesthesia was used, but MBD is thought to be possible under local anesthesia [23].

Postoperative bleeding due to blunt dissection may be a concern, but with adequate infiltration of 1 % lidocaine + epinephrine (3-fold dilution), little postoperative bleeding was noted with mild postoperative compression with absorbent gauze.

Dissemination of cancer cells by tearing the tumor may be considered when applying blunt dissection, so our surgical technique is not indicated in patients with suspected tumor infiltration into the skin. However, because a subcutaneous skin flap with appropriate layers can be created with minimal sharp dissection, there is little or no risk of cutting into the tumor. Surgeons at our institution have been performing this surgical procedure since 2004, and no local recurrence due to our dissection technique has occurred. Long-term evaluation in a larger number of cases, however, would be desirable.

## Conclusions

In conclusion, our proposed technique for partial mastectomy using MBD provides good curative and cosmetic results.

## Abbreviations

Bp: Breast-preserving surgery
SNB: Sentinel lymph node biopsy
MBD: Manual blunt dissection
MRI: Magnetic resonance imaging
DCIS: Ductal carcinoma in situ
SN: Sentinel lymph node
RI: Radioisotope
LTF: Lateral tissue flap
LDMF: Latissimus dorsi muscle flap.
